# Coronary artery calcification score as a prognostic factor in younger patients with multiple myeloma undergoing autologous stem cell therapy

**DOI:** 10.1186/s40959-025-00338-1

**Published:** 2025-04-23

**Authors:** Hans-Jonas Meyer, Wolfram Pönisch, Jan Borggrefe, Alexey Surov

**Affiliations:** 1 Department of Diagnostic and Interventional Radiology, University of Leipzig, 04103 Leipzig, Germany; 2 Department of Hematology and Cell Therapy, University of Leipzig, Leipzig, Germany; 3 Department of Radiology and Nuclear Medicine, Muehlenkreiskliniken Minden, Ruhr-University of Bochum, Bochum, Germany

**Keywords:** CT, CAC score, Multiple myeloma

## Abstract

**Background:**

Coronary artery calcification (CAC) scoring can be performed as a by-product of computed tomography (CT). CAC scoring may reflect the general cardiovascular risk profile of patients. The aim of the present study was to determine the impact of CAC on overall survival (OS) in patients with multiple myeloma (MM).

**Methods:**

A retrospective analysis was conducted on all patients with MM undergoing peripheral blood stem cell transplantation between the years 2009 and 2019. A total of 127 patients (50 female patients, 39.4%) with a mean age of 57.8 ± 7.6 years were included in the analysis. A whole-body CT scan was used to assess the CAC score for each patient. The Weston score as a surrogate for Agatston score was applied in the non-gated staging CT images.c

**Results:**

A total of 27 patients (22.0%) died during the course of the study. The CAC score did not differ between non-survivors and survivors in the discrimination analysis (mean 1.2 ± 2.4 versus 2.0 ± 2.8, *p* = 0.13). The CAC score showed no correlation with overall survival, with an HR of 0.92 (95% CI 0.78–1.09, *p* = 0.35). Of the patients without calcification (CAC score 0, *n* = 66, 51.9%), 18 died, while of those with calcification (CAC score 1 or higher, *n* = 61, 48.1%), nine died. The results of the Fisher’s exact test showed no statistically significant difference between the two groups (*p* = 0.20).

**Conclusions:**

The presence of CT-defined coronary calcifications does not predict survival in younger patients with multiple myeloma undergoing autologous stem cell therapy and comparably short survival. The impact of CT-defined cardiovascular risk factors appears to be relatively modest in this heterogeneous disease.

## Background

Computed tomography (CT)-defined coronary artery calcium (CAC) scoring is a widely used biomarker to quantify the burden of calcified plaque in the coronary arteries. This is of significant prognostic importance in patients with coronary artery disease (CAD) [1–4]. A strong correlation has been demonstrated between CAC scores and the occurrence of major cardiovascular events in asymptomatic individuals. It has also been shown to be an effective predictor of overall vascular health.

In general, the widely used Agatston score is calculated on cardiac-gated CT images [4], whereas the more recently developed Weston score is calculated semiquantitatively on non-gated CT images and can therefore be calculated on staging CT images [5, 6].

Despite the clear published benefits of CAC scoring in cardiology, there are also promising results regarding the prognostic relevance of CAC in oncology patients [7–9]. In particular, several studies have investigated the prognostic implications of CAC in lung cancer patients [9, 10].

In multiple myeloma (MM), CAC scoring may be of particular interest as this disease predominantly affects elderly patients with a median age of 70 years, resulting in a high frequency of cardiovascular comorbidities [11]. However, the effect of CT-defined CAC scoring has not been investigated in MM patients in a systematic manner.

The aim of the present study was to determine whether CT-derived computed tomography CAC scoring is a prognostic indicator for overall survival in patients with MM undergoing autologous peripheral blood stem cell transplantation (aPBSCT).

## Methods

### Patient acquisition

This retrospective observational study was approved by the institutional review board (IRB 00001750; registration number 118/18-ek).

All patients with MM who underwent autologous PBSCT with curative intent between 2009 and 2019 were retrospectively included in the study. Bortezomib was used as an induction therapy throughout the time period.

A total of 127 patients (50 female patients, 39.4%) with a mean age of 57.8 ± 7.6 years with sufficient clinical and imaging data for analysis were identified. No patient was excluded due to inadequate CT image quality.

### Clinical parameters

The following clinical parameters were obtained from the patients’ medical records: blood count; serum levels of C-reactive protein, lactate dehydrogenase (LDH), b2-microglobulin, creatinine, albumin and calcium; creatinine clearance as a factor of renal insufficiency; quality and quantity of M protein in blood and urine samples; clinical stage according to the Salmon and Durie classification and the International Staging System (ISS); cytogenetics: Autologous peripheral blood stem cell transplantation (aPBSCT); the assessment of disease progression was conducted in accordance with the International Myeloma Working Group guidelines [12]; death and overall survival.

### Imaging technique

CT imaging was performed on a clinically used 128-slice or 256-slice CT scanner (Ingenuity or iCT256, Philips, Hamburg, Germany). The CT scan at the time of diagnosis was used to calculate the CAC score. The imaging parameters used were 120 kVp, 36 mAs, 64 × 0.6 mm collimation and 0.8 pitch. The scan length included the following body regions: head, neck, chest, abdomen/pelvis, upper and proximal half of the lower extremity. The minimum slice thickness was 1 mm.

### CAC scoring

**Fig. 1:**
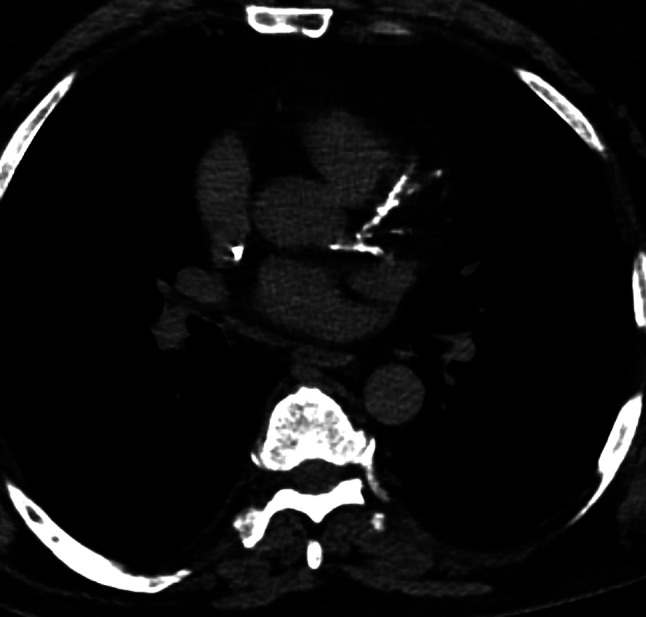
Representative male patient of the patient sample. One can appreciate extensive calcifications in all coronary vessels with a resulting CAC score of 10

The Weston scoring was performed by an experienced radiologist with seven years of experience in cardiovascular radiology. The extent of calcification in the four coronary arteries (left main, left anterior descending, right coronary and left circumflex) was scored on a 4-point scale Scores of 0, 1, 2, and 3 were assigned based on the presence and extent of calcium. A score of 0 indicated the absence of calcium, while a score of 1 indicated the presence of a single pixel of calcium. A score of 2 indicated the presence of multiple pixels of calcium, but not sufficient for classification as a score of 3. A score of 3 indicated the presence of “hard” calcium, as evidenced by blooming artifacts, as previously described [13]. The total Weston score is the sum of the four arteries ranging from 0 to 12 [13]. In the initial description of the score, a strong correlation was observed with the Agatston score (*r* = 0.83, *p* < 0.0001), with a low interreader variability for both cardiac-specialized radiologists and non-specialized radiologists [13]. Figure 1 depicts a representative patient from the study, illustrating the CAC score.

### Statistical analysis

The statistical analysis and graph generation were conducted using GraphPad Prism 10 (GraphPad Software, La Jolla, CA, USA) and the SPSS package (IBM SPSS Statistics for Windows, version 225.0: IBM Corporation). The data were expressed using descriptive statistics including absolute and relative frequencies. Spearman’s correlation coefficient (r) was employed to examine the associations between CAC score and clinical parameters. The Mann-Whitney-U test and the Fisher’s exact test were used to test for group differences when appropriate. A binary logistic regression analysis was used for associations between CAC score and overall survival. Kaplan-Meier curves were used to visualize the survival times. In all tests, a p-value of less than 0.05 was considered statistically significant.

A power calculation was performed with the published mean value of the CAC 2.53 ± 3.48 in a recent publication of unselected patients in the emergency department [14]. With the estimation of a mean CAC score of 1.2 in a patient cohort with hematological disorders, the calculated patient sample to draw statistically significant conclusions was 54 with a statistical power of 80%.

## Results

A total of 27 patients (22.0%) of the patient sample died; median 33 months (95% CI 28;40), range 4-396 months. The median follow-up time was 49 months. All cases died of MM-related causes.

**Fig. 2:**
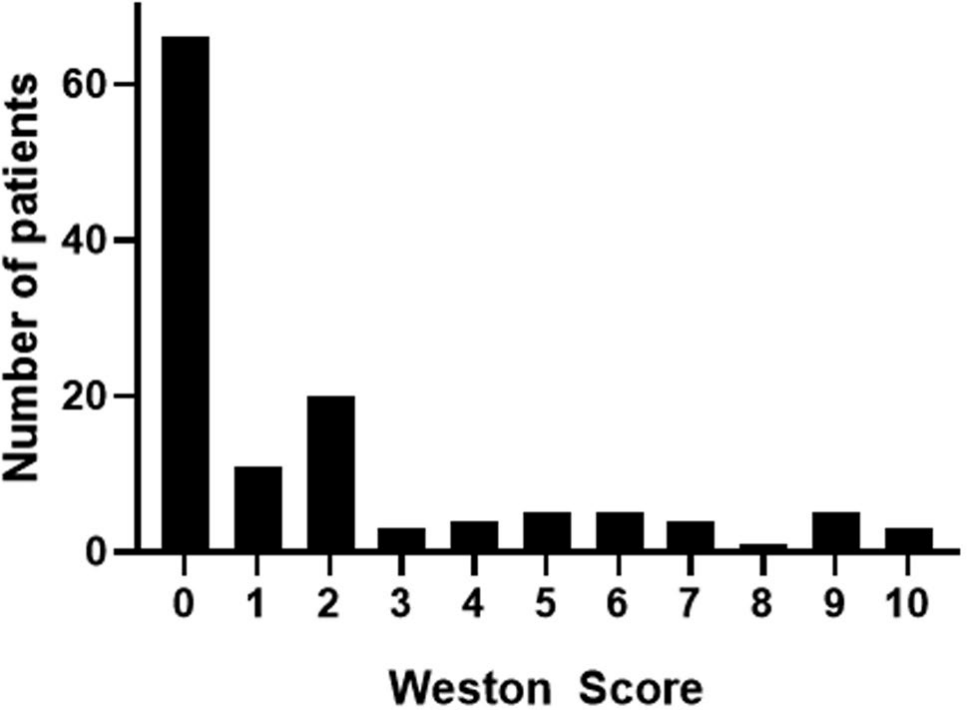
Histogram distribution of the CAC score of the patient cohort. Over half of patients had no visible calcifications (CAC score 0, 51.9%)

Table 1 summarizes the demographics of the patient sample. The mean CAC score was 1.9 ± 2.8, ranging from 0 to 10. A total of 66 patients (51.9% of the patient sample) had no calcifications (CAC score 0). Figure 2 provides the CAC score distribution of the patient cohort. The CAC score was significantly higher in male patients compared to female patients (mean 2.6 ± 3.0 versus 0.80 ± 2.0, *p* < 0.0001).

In three cases, cardiac amyloidosis was the cause of death. Of these, 2 cases had a CAC-score of 0 and one case had a CAC score of 3.

**Table 1:** Baseline characteristics, treatments, and causes of death. The patient sample was comprised of 127 patients

Parameter	mean ± SD;
Age (y)	57.8 ± 7.6
Gender (female, n, %)	50 (39.4%)
Albumin (g/dL)	42.1 ± 32.3
Calcium (mmol/L)	2.4 ± 0.3
Beta-microglobulin (mg/L)	7.9 ± 19.2
ISS stage (n, %)	I: 54 (42.5%) II: 35 (27.6%) III: 38 (29.9%)
Salmon and Durie stage (n, %)	Ia: 2 (1.6%) IIa: 6 (4.7%) IIIa: 89 (70.0%) IIIb: 30 (23.7%)
CAC score	1.9 ± 2.8
No coronary calcification (CAC score 0)	66 (51.9)

Abbreviations: SD = standard deviation, ISS = International Staging system; CAC = coronary artery calcification

### Survival analysis

The CAC score did not differ between non-survivors and survivors in the discrimination analysis (mean 1.2 ± 2.4 versus 2.0 ± 2.8, *p* = 0.13).

In the univariable logistic regression analysis, the CAC score showed no correlation with overall survival, with an HR of 0.92 (95% CI: 0.78–1.09), p-value = 0.35. In addition, no associations between CAC score and PFS were found, HR = 0.88, 95%CI (0.70;1.10), *p* = 0.27. As no meaningful results were shown in the univariable analysis, a multivariable analysis with other potential prognostic factors including age and ISS stage was omitted.

A total of 18 patients died in the group without calcification (CAC score 0, *n* = 66, 51.9%), while nine patients died in the group with calcification (CAC score 1 or higher, *n* = 61, 48.1%). The Fisher’s exact test yielded no statistically significant difference between the two groups (*p* = 0.20).

**Fig. 3:**
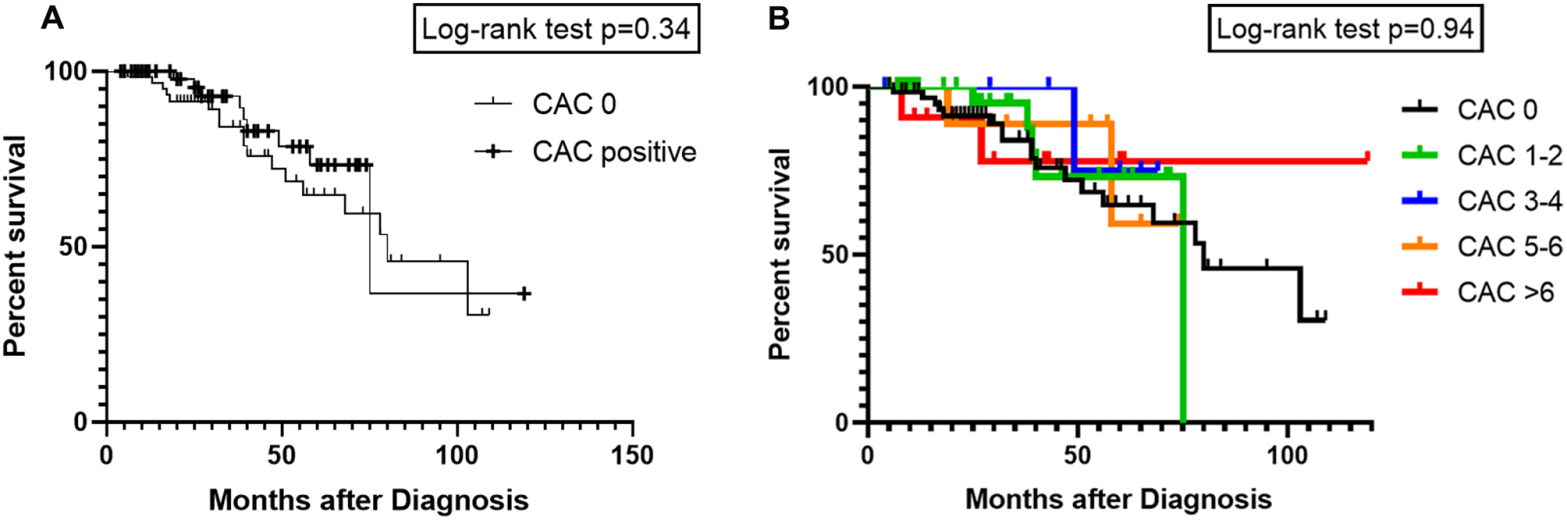
**(A)** Kaplan-Meier curves for the overall survival comparison between patients with calcifications (CAC score 1 and higher) versus patients without calcifications (CAC score 0). The overall survival was not different between the groups (*p* = 0.34). **(B)** Kaplan-Meier curves with stratifications for calcification groups in CAC score 1–2, CAC score 3–4, CAC score 5–6 and CAC score above 6. The overall survival was not different between the groups (*p* = 0.94)

In the Kaplan-Meier curves, similar results were shown (Fig. 3). The median survival for the group without calcifications (CAC score of 0) was 80 months, compared to the group with calcifications was 75 months, *p* = 0.34 (Fig. 3a). After stratification of the patients with calcifications, there was also no statistically significant difference between the groups (*p* = 0.94, Fig. 3b).

### Subgroup analysis according to gender

In the male patient group, there were 19 lethal cases (24.7%). In the patient group without calcifications (CAC score 0, *n* = 29), there were 10 lethal cases (34.5%), and in the patient group with calcifications (CAC score 1 or higher, *n* = 48), there were 9 lethal cases (18.8%). The Fisher’s exact test yielded no statistically significant difference between the two groups (*p* = 0.29).

In logistic regression analysis, there was no association with the overall survival with an HR of 0.87 (95%CI 0.71;1.07, *p* = 0.19).

The CAC score did not differ between lethal and non-lethal cases (mean 1.8 ± 2.7 versus 2.8 ± 3.1, *p* = 0.15).

In the female patient group, there were 8 lethal cases (16%). In the patient group without calcifications (CAC score 0, *n* = 37), there were 8 lethal cases (21.6%), and in the group with calcifications (*n* = 13), there were 0 lethal cases. The Fisher’s exact test yielded no statistically significant difference between the two groups (*p* = 0.17).

In logistic regression analysis, there was no association with the overall survival with an HR of 0.98 (95%CI 0.97;1.1, *p* = 0.89).

The CAC score did not differ between lethal and non-lethal cases (mean 0 versus 0.9 ± 2.2, *p* = 0.11).

## Discussion

The present study sought to investigate the prognostic relevance of the CAC score defined by CT images on overall survival in patients with MM undergoing autologous PBSCT.

In conclusion, the study found no association between coronary calcification and survival outcomes in patients with MM.

CAC scoring is an extensively studied imaging biomarker, most notably in the context of the Framingham Heart Study [1–4, 15]. Since then, the prognostic significance of coronary artery calcified plaque burden in patients with coronary artery disease has been clearly established and translated into clinical practice [1–4]. Furthermore, CAC scoring is an effective predictor of overall vascular status and has been demonstrated to possess prognostic relevance in numerous disease states [1–4, 16, 17].

Until recently, cardiac-gated CT studies were the only method available for quantifying coronary artery calcium. The increased number of detectors and faster gantry speeds in current CT scanners permit the use of non-gated studies for the purpose of providing either semiquantitative (ordinal scores) or quantitative CAC (Agatston scoring). These studies demonstrated a high correlation between semiquantitative scores like the Weston score with cardiac-gated CT studies and most notably cardiovascular disease outcomes [4, 6, 13].

Accordingly, it is appropriate to utilize the diagnostic staging CT images used to detect myeloma manifestations also to calculate the CAC score in a clinical setting.

Nevertheless, despite its high prevalence in clinically asymptomatic patients and the high frequency of staging CT investigations, the prognostic relevance of CAC scoring remains a topic that has only been explored in a limited number of studies within the field of oncology.

There is ongoing research for development and evaluation of artificial intelligence algorithms for calculating the Agatston score from non-cardiac gated staging CT scans. This could potentially enhance clinical care in the near future, once these scans become widely accessible [18].

In a PET-CT study investigating a heterogenous oncological patient sample, CAC was associated with a combined study endpoint of death and cardiovascular events in both univariable and multivariable analysis (OR 2.6 (95% CI 1.42–4.77) (*p* < 0.01) [19]. In a multicenter study on diffuse large B-cell lymphoma, the CAC score was automatically measured in overall 1,468 patients [20]. In this large cohort, a highly significant association of the coronary artery calcium score with major cardiovascular events was found [20]. The primary objective of this study was to identify high-risk patients who may experience cancer therapy-related cardiac dysfunction and major adverse cardiac events. This information will assist clinicians in implementing appropriate cardiovascular protection strategies for patients with diffuse large B-cell lymphoma who have received anthracycline-based chemotherapy.

When comparing the results of the different studies and their results, it should be emphasized that the measurement method of the coronary calcification could have an influence on the results. The present semiquantitative Weston score may provide less information compared with the more advanced algorithm of the study by Shen et al. [20]. Information regarding the Hounsfield density of the coronary plaques, which is included in the algorithm is not included into the Weston score method. Moreover, the Weston score is only a surrogate method to quantify the exact plaque burden, which is better measured by the quantitative algorithm method.

Therefore, it remains uncertainty, if the used CAC measurement has also an influence on the negative results of the present study. There is need for another study using also an advanced algorithm to measure the coronary calcifications in patients with MM.

In another study, the CAC score was employed in patients with acute myeloid leukemia, with the objective of guiding patients with higher cardiac risk [21]. In patients with lung cancer, the CAC score may assist in the prediction of major adverse cardiovascular events in those with curable disease [9].

One important aspect for patients with MM is the high patient age, with a median age of 70 years, which should result in a high prevalence of cardiovascular disease [11]. However, the mean age of the cohort under investigation for MM is younger, with a mean age of 57.8 years, largely due to the aggressive treatment form of autologous PBSCT and therefore possible selection bias. This may be a significant factor contributing to the inverse correlation between CAC and OS observed in this younger cohort. The risk for a selection bias should be considered, when interpreting the present results.

It is also important to note that, even in this younger patient cohort, there was a relatively high frequency of coronary calcifications, with 48.1% of patients exhibiting this finding.

Furthermore, it is possible that treatment may have cardiotoxic effects or even directly damage the heart in patients with MM [11]. Our findings revealed a notable prevalence of coronary calcifications in male patients, yet this did not result in discernible differences in survival analysis. Therefore, the elevated cardiovascular risk profile observed in male patients was not associated with outcomes in MM patients. Nevertheless, the impact of MM on CAC score appears to be minimal and could not be demonstrated to have a significant effect in the cohort under investigation.

One can only hypothesize why the CAC score has less impact in this tumor entity compared to other tumors, such as lung cancer. First, it is a more heterogeneous tumor with different biological behaviours and disease manifestations. This could have an influence on the investigated cohort with potential selection bias. It may be that the effect of CAC score as a surrogate for the cardiovascular risk may be less important in patients with MM. However, it has been demonstrated that the treatment regimes for MM have a relevant cardiotoxicity profile inducing atrial fibrillation, arterial hypertension, and cardiac ischemia [22]. In addition, there is strong evidence for the cardiotoxicity induced by stem cell transplantation treatment, which can be stratified by short term effects caused by treatment conditioning regimen and graft-versus host effects and then by long term effects with a risk profile for coronary artery disease [23, 24]. The reported incidence of acute heart failure ranges from 0.4 to 2.2% [24]. These results demonstrate the high importance of cardiovascular risk management and surveillance. Further studies are needed to elucidate the potential role of CT-defined coronary calcifications as a risk factor in these patients, despite the negative results in the current study.

Clearly, there is more research needed in the field of cardio-oncology to elucidate the complex interactions between CAC score as a cardiovascular risk imaging marker and survival outcomes in haematological diseases.

It is evident that a larger patient cohort investigated in a multicenter setting is required to provide more robust and higher-quality evidence regarding the prognostic role of CAC scoring in patients with MM.

There are limitations of the present study to address. First, it should be noted that the retrospective observational study design may be susceptible to potential bias. Second, the sample size is relatively limited due to the single-center design. We provided a power calculation to demonstrate that the current sample size is sufficiently powered to show distinctive difference of the CAC scoring of hematological patients. Moreover, the current study included every available case in our hospital and could not provide a more comprehensive analysis. Third, CAC scoring was conducted using a non-cardiac gated CT. However, there is sufficient data that the Weston score used has a very high correlation with the gold standard Agatston score (*r* = 0.83) [13]. Beyond that, there may be certain reader induced bias. However, in the first description of the Weston score, the interreader agreement was very high with a reported intraclass coefficient of 0.93 [13], which was also reproduced in another study with a reported intraclass coefficient of 0.92 [14]. The risk of a potential reader bias for the current study seems therefore to be very low. Fourth, an investigation into the potential occurrence of major cardiovascular events in the survivor cohort was not possible due to a lack of clinical assessment. Further analyses are required to demonstrate the predictive value of CAC scoring in long-surviving MM patients for major cardiovascular events. A fifth potential explanation for the observed negative associations between CAC scoring and OS is the relatively short life span until the fatal outcome in the present cohort. It seems reasonable to posit that the prognostic effect of coronary calcifications is likely to be more pronounced in oncological patients with a longer life span, as evidenced by other studies [25].

## Conclusions

The presence of CT-defined coronary calcifications does not correlate with survival outcomes in younger patients with MM undergoing autologous stem cell therapy and experiencing progression of disease by MM. The impact of CT-defined cardiovascular risk factors appears to be relatively limited in this heterogeneous disease, despite their high prevalence, observed in 48.1% of patients.

## Data Availability

No datasets were generated or analysed during the current study.

## Abbreviations

CT: Computed tomography
MM: Multiple myeloma
aPBSCT: Autologous stem-cell therapy
HR: Hazard ratio
ISS: International Staging system
